# Retracted: *Crocus sativus* L. (Saffron) Stigma Aqueous Extract Induces Apoptosis in Alveolar Human Lung Cancer Cells through Caspase-Dependent Pathways Activation

**DOI:** 10.1155/2020/3516010

**Published:** 2020-10-08

**Authors:** BioMed Research International

In the article titled “*Crocus sativus* L. (Saffron) Stigma Aqueous Extract Induces Apoptosis in Alveolar Human Lung Cancer Cells through Caspase-Dependent Pathways Activation” [1] concerns were identified with figure duplication, as originally noted on PubPeer.

Figure elements in each panel of Figure 2 and in Figure 7B display indications of duplication. The authors were unable to provide the original high-resolution underlying images for Figures 2 and 7, any replicates, and the individual data points behind Table 1 and Figures 1 and 4 due to a laptop failure. The Editorial Board originally agreed to allow the authors to attempt to replicate this work under the supervision of their institution.

An expression of concern was published while the replication attempt and investigation continued. Following the publication of this notice, additional overlap wad identified between Figure 2 [1] Figure 2 from a previous publication [2].

The journal and the handling editor are, therefore, retracting this article due to concerns with the validity of the results. The authors do not agree to the retraction.
